# The angiosperm radiation revisited, an ecological explanation for Darwin’s ‘abominable mystery’

**DOI:** 10.1111/j.1461-0248.2009.01342.x

**Published:** 2009-09

**Authors:** Frank Berendse, Marten Scheffer

**Affiliations:** 1 Nature Conservation and Plant Ecology Group, Wageningen UniversityPO Box 47, 6700 AA Wageningen, The Netherlands; 2 Aquatic Ecology and Water Quality Group, Wageningen UniversityPO Box 47, 6700 AA Wageningen, The Netherlands

**Keywords:** Angiosperms, bogs, evolutionary radiation, gymnosperms, heathlands, plant–soil feedbacks

## Abstract

One of the greatest terrestrial radiations is the diversification of the flowering plants (Angiospermae) in the Cretaceous period. Early angiosperms appear to have been limited to disturbed, aquatic or extremely dry sites, suggesting that they were suppressed in most other places by the gymnosperms that still dominated the plant world. However, fossil evidence suggests that by the end of the Cretaceous the angiosperms had spectacularly taken over the dominant position from the gymnosperms around the globe. Here, we suggest an ecological explanation for their escape from their subordinate position relative to gymnosperms and ferns. We propose that angiosperms due to their higher growth rates profit more rapidly from increased nutrient supply than gymnosperms, whereas at the same time angiosperms promote soil nutrient release by producing litter that is more easily decomposed. This positive feedback may have resulted in a runaway process once angiosperms had reached a certain abundance. Evidence for the possibility of such a critical transition to angiosperm dominance comes from recent work on large scale vegetation shifts, linking long-term field observations, large scale experiments and the use of simulation models.

## Introduction

The angiosperm radiation is one of the evolutionary events that most puzzled Darwin. In fact, he famously referred to it as an ‘abominable mystery’. The seemingly sudden appearance of so many angiosperm species in the Upper Chalk conflicted strongly with his gradualist perspective on evolutionary change (Friedman 2009). Obviously, since Darwin many new fossils have been found and recently molecular data have given new opportunities to unravel evolutionary patterns (Doyle 2001). We now know that the major groups, including the Chloranthaceae, the magnolids, eudicots and monocots, already originated in the Early Cretaceous, *c*. 125 million years ago (Ma), but there is no convincing evidence of angiosperms from pre-Cretaceous periods (Doyle & Hickey 1976; Crane *et al.* 1995; Doyle 2001; Friis *et al.* 2006). The emerging picture is that after the divergence of the major angiosperm lines by the late Barremian (125 Ma), there was a steady, but impressive increase in diversity of angiosperms (Crane *et al.* 1995; Doyle 2001; Friis *et al.* 2006). In many speculations on the reasons for the great success of angiosperms there has been some confusion about their success in terms of species diversity (Crepet & Niklas 2009), and their success in terms of abundance and ecological dominance. Nevertheless, nowadays most authors assume that during the first millions of years angiosperms remained relatively rare until eventually an impressive rise to ecological dominance took place (Wolfe 1997). In the Albian (105 Ma) the percentage of angiosperms in local paleofloras was still only 5–20% but this percentage had increased to 80–100% in the Maastrichtian at the end of the Cretaceous (65 Ma) (Crane & Lidgard 1990). Lupia *et al.* (1999) analysed a database of North American palynological samples from Cretaceous sediments and concluded that at middle latitudes the diversification of angiosperms was almost matched by their increasing abundance with a time lag of *c*. 10 Ma. After the angiosperms had entered the fossil record at low to middle latitudes, the spread of the angiosperms poleward occurred during the medial and Late Cretaceous (Wolfe 1997), although some authors argued that angiosperms did not become dominant until the Early Tertiary (Wing & Boucher 1998). Following the events at the Cretaceous-Tertiary boundary (65 Ma) many angiosperm lines, comprising a large part of the extant eudicot groups, showed a rapid diversification at the genus and even family level replacing some of the old lineages and filling empty niches (Wolfe 1997). Recently, Magallón & Castillo (2009) showed that the more recent angiosperm lineages (comprising more than half of the living species) have high diversification rates as compared with the older lineages that originated in the Early Cretaceous. Attempts to explain the stunning diversification and the rise to ecological dominance of angiosperms have stressed the importance of key innovations such as pollination and seed dispersal by animals or their fast-growing and rhizomatous growth form (Regal 1977; Mulcahy 1979; Doyle & Donoghue 1993). However, there does not seem to be a simple set of biological features that was key to their impressive success (Davies *et al.* 2004).

Another line of work has tried to illuminate the ecology of early angiosperms. This produced a range of hypotheses, including the idea that early angiosperms were:

1. Weedy shrubs that lived in open, disturbed habitats of semi-arid tropical to subtropical regions (Axelrod 1970).
2. Plants of disturbed streamside habitats in mesic environments (Hickey & Doyle 1977).
3. Fast-growing, semi-herbaceous plants of sunny, unstable streamsides that tolerated disturbance and had high leaf photosynthetic capacity and short generation times (Wing & Boucher 1998).
4. Woody plants that grew in dimly lit, disturbed forest understorey habitats and shady streamside settings (Feild *et al.* 2004).
5. Fragile, herbaceous plants growing in aquatic of marsh habitats (Hill 1996; Sun *et al.* 2008).
6. Small plants with xeromorphic features suggesting that they grew in dry environments (Mohr & Rydin 2002; but see Doyle *et al.* 2008; Mohr & Friis 2000; Mohr & Eklund 2003).

Early angiosperms were apparently most frequently found in sites that were assumed to be disturbed, aquatic or extremely dry. The only overlapping aspect of these views is that early angiosperms were assumed to grow in sites without ferns or tall gymnosperm trees. In our opinion, this suggests that they had difficulties competing with these groups of plant species. Yet at the end of the Cretaceous (65 Ma) angiosperms had become major contributors to biomass not only in disturbed or aquatic habitats, but also in stable terrestrial environments, where competition with gymnosperms and ferns must have played a significant role (Hickey & Doyle 1977). The idea that direct competition between the groups was important is in line with indications that gymnosperms were eventually affected by the rise of angiosperms. The dominant gymnosperms in the tropics, the Cheirolepidiaceae, went extinct rather soon after the first angiosperm radiation (Alvin 1982). At the beginning of the Cretaceous, forests were dominated by tall conifer trees, but during the Aptian and Albian (125–100 Ma) the conifers and many other gymnosperms declined (Wolfe 1997) and by the end of the Cretaceous a large variety of gymnosperm groups (Bennettitales, *Eucommiidites*, *Baiera* of the Ginkgoales and several conifer groups) had disappeared (Stewart & Rothwell 1993). But Lupia *et al.* (1999) using palynological data for North America concluded that during the Cretaceous gymnosperm abundances declined only slightly at mid-latitudes, while free-sporing plants, mostly ferns, decreased more markedly. We assume that at some time during the Cretaceous angiosperms entered the fern-dominated understorey of tall conifer forests, explaining this decline of ferns, while the decline of some conifer groups occurred later when also angiosperm trees started to play a prominent role. Nowadays lowland gymnosperm forests at mid- and low latitudes have been replaced completely by angiosperm-dominated woodlands.

To us the most puzzling aspect of this massive take-over is that after millions of years of apparent suppression by gymnosperms, the angiosperms at some point escaped from their subordinate position to replace the gymnosperms and ferns and become dominant around much of the globe. What made the competitive balance between these groups tip at some point in time?

## An ecological explanation for the shift towards angiosperm dominance

**Figure 1:**
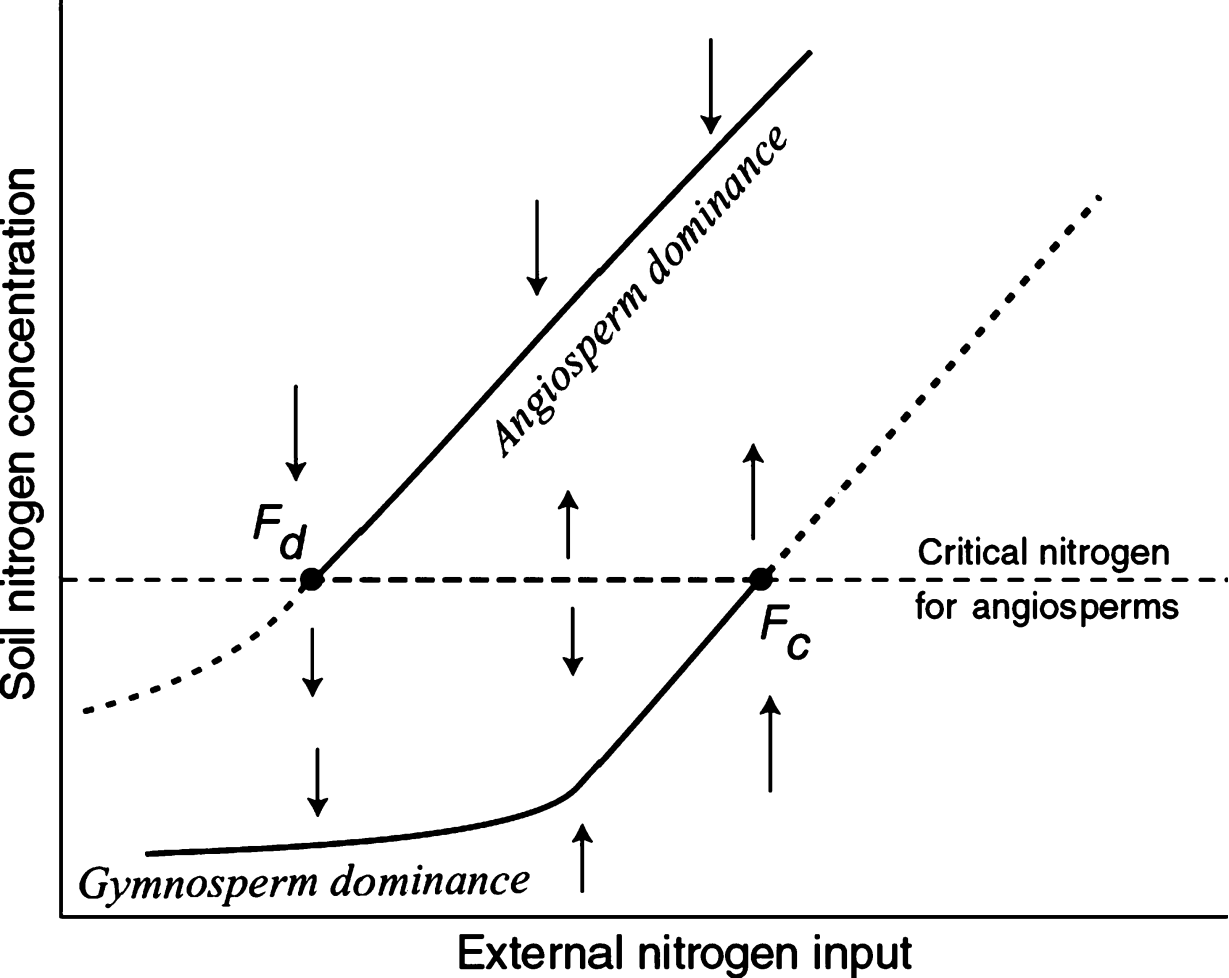
A simple graphical model illustrates more specifically how competition between angiosperms and gymnosperms might work out. The model is based on two assumptions: (1) given a certain nitrogen input, soil inorganic N-concentrations will be lower in a system with gymnosperm dominance than in a system with angiosperms and (2) angiosperms need a critical N-supply for growth that is higher than the supply needed by gymnosperms. The emerging picture shows that over a range of intermediate global nitrogen levels (and hence local nitrogen inputs) two alternative equilibria exist: one dominated by angiosperms and one dominated by gymnosperms. At lower nutrient levels, only the gymnosperm-dominated equilibrium exists, whereas at the highest nutrient levels, there is only an angiosperm-dominated equilibrium. If we suppose that the overall nitrogen level of the Earth was in the range where alternative equilibria exist (between the bifurcation points *F*_*d*_ and *F*_*c*_), early angiosperms would not be able to invade a gymnosperm dominated world except on relatively nutrient rich disturbed sites where the soil nutrient concentrations exceed the critical level needed by this group. The arrows indicate the direction of change when the system is not in one of the two alternative stable states, illustrating that only both solid lines represent stable equilibria (Scheffer *et al.* 2001).

Here, we propose that the massive scale of the shift in Earth vegetation composition may be explained as the result of a positive feedback between angiosperms and their environment. Although factors limiting plant growth probably varied strongly among the many different habitats (as they do nowadays) we assume that soil nutrients, especially N and P (Verhoeven *et al.* 1996), and water have been the major growth limiting factors. During the Cretaceous CO_2_-levels in the atmosphere were several times higher than today resulting in increased stomatal closure and reduced water stress (Barrett & Willis 2001), which probably reinforced the important role of soil nutrients. We argue that angiosperms have higher growth rates and need higher nutrient levels than the gymnosperms that dominated before, whereas at the same time angiosperms promote soil nutrient levels by producing litter that is more readily decomposed. In this view, the prolonged dominance by gymnosperms could reflect a classical example of hysteresis ending in a rapid shift to an alternative stable state (Fig. 1; Scheffer *et al.* 2001). The conditions under which the co-existence of competing species (or groups) is unstable are well known from classical competition theory. The general rule illustrated by Lotka-Volterra models is that competition can lead to unstable co-existence (implying alternative stable states) if it is more favourable to have conspecifics around than individuals of the other species. As we will show, this may well characterize the competition between gymnosperms and angiosperms. We hypothesize that gymnosperms do relatively well under low nutrient conditions, and also maintain low nutrient levels in the soil due to the nature of their litter. Angiosperms do not grow well under such conditions but once they are present in sufficient densities they enhance soil fertility through their litter implying a positive feedback that might produce a runaway process once angiosperms have reached a certain critical abundance.

The observation that early angiosperms occurred largely at disturbed and at xeric or aquatic sites would be well in line with our hypothesis. In all these sites, we might expect relatively little competition from gymnosperms and ferns. The weedy, fast-growing habit of many early angiosperms enabled them to spread rapidly in bare but unstable environments, such as tidal flats (Retallack & Dilcher 1981) and fresh sand deposits along streams and rivers (Doyle & Hickey 1976; Hickey & Doyle 1977). Probably, later, after further diversification, angiosperms were able to enter the understorey of coniferous forests, most likely using disturbed sites as a starting point. Disturbances through fires, storms or huge dinosaurs trampling, grazing and pushing down complete trees created gaps in existing stands of tall conifers. In such gaps, competing plants were removed while nutrient supply to the plant was increased. Disturbance of forest soils typically leads to increased decomposition and nutrient release from the dead organic matter (Matson & Boone 1984; Kristensen *et al.* 2000). Bond (1989) with his ‘slow seedling’ hypothesis proposed that in such gaps the fast growing angiosperm seedlings were competitively superior to the slow seedlings of the conifers and other gymnosperms, eventually explaining the large scale decline of the gymnosperms as a result of the invasion of angiosperms in the gymnosperm regeneration niche.

What is the evidence that gymnosperms may have effectively suppressed angiosperms initially? The implications of dominance of extant gymnosperm species for soil fertility are well established. Gymnosperms generally have longer leaf life spans and higher lignin concentrations and lower N and P concentrations in their leaves (Grime *et al.* 2007; Cornwell *et al.* 2008). Longer leaf life spans increase the carbon assimilation per unit of absorbed nutrient and reduce the relative nutrient losses from the plant. Model simulations show that such plant features are favoured in nutrient-poor environments if plants are subject to interspecific competition (Berendse 1994a). In line with this, litter decomposability in recent gymnosperm species is generally much lower than in angiosperms. In an overview of 66 experiments including 818 species, Cornwell *et al.* (2008) found that on average gymnosperm litter decomposed *c*. 60% slower than the litter of eudicots. It is common knowledge that these differences have enormous consequences for the dynamics of dead organic matter in forest soils. Modern gymnosperm forests generally produce morr soils with a clear fermentation-humus layer and only little fragmenting activities of soil animals, while angiosperm trees produce mull soils with a high activity of decomposing soil animals and a high degree of mixing of dead organic matter and mineral soil particles (Müller 1879).

While the fact that gymnosperms tend to create poorer soils is an essential ingredient of our hypothesis, it is not a sufficient condition to create alternative stable states. The complementary necessary ingredient is that angiosperms require and create higher soil nutrient levels than gymnosperms. Also, it is not sufficient to demonstrate the existence of positive-feedback mechanisms to prove that alternative stable states can exist. The strongest cases for the occurrence of this system property are built by combining such insights not only with field observations but also with experiments on relevant scales (Scheffer & Carpenter 2003). Obviously, this is not feasible if it comes to explaining massive events in deep time. Nonetheless, as we will show, recent shifts in vegetation composition may serve as analogues to support our view of the rise to dominance of the angiosperms during the Cretaceous and subsequent periods. Work on recent shifts has led to a thorough mechanistic understanding by linking the use of models, experiments and analysis of field patterns.

## Positive feedbacks and sudden vegetation shifts in heathlands

As a central example to illustrate our line of thought we consider the case of shifting heathland vegetations. Many heathlands in Western Europe changed during the last 30 years from dwarf-shrub-dominated communities to monospecific stands of perennial grasses (de Smidt 1995). This change was triggered by increased atmospheric N-deposition and the accumulation of soil organic matter after abandonment by farmers, who used the heathlands for sheep grazing and turf cutting (Berendse 1990). The higher N-deposition accelerated the accumulation of soil organic matter and strongly enhanced soil nitrogen mineralization. This increase in nutrient supply had far reaching consequences for the competitive balance among the dominant plant species.

Like gymnosperms, ericaceous dwarf shrubs, such as *Erica tetralix*, *Calluna vulgaris* and *Vaccinium vitis-idaea*, have leaves and stems with long life spans, which enable them to minimize nutrient losses but also results – as a negative side effect – in very low potential growth rates due to the high biosynthesis costs of these tissues (Table 1). In contrast, the perennial grasses that have expanded during recent years, such as *Molinia caerulea* and *Dechampsia flexuosa*, have leaves with much shorter life spans, but also much higher potential growth rates. Several experiments have shown that the slow growing, economical species were able to maintain dominance at low soil nutrient levels, but after the initial increase in nutrient availability the grasses rapidly increased and replaced the dwarf shrubs (Heil & Diemont 1984; Aerts & Berendse 1988).

**Table 1:** Relative biomass turn-over, potential growth rate (as measured in field fertilization experiments) and weighted litter mass loss rates (for leaves, stems, flowers and roots) of *Erica tetralix* and *Molinia caerulea* (Berendse 1994b;Van Vuuren *et al.* 1993)

	Erica	Molinia
Leaf life span (year)	1.3	0.35
Lignin concentration (%)	33	24
Biosynthesis costs (g glucose per g biomass)	1.8	1.4
Relative biomass loss rate (g g^−1^ year^−1^)	0.58	1.48
Potential growth rate (g m^−2^ year^−1^)	769	> 1794
Litter decomposition rate (g g^−1^ year^−1^)	0.06–0.09	0.24–0.26

The longer leaf life spans of dwarf-shrub leaves are made possible by their greater toughness and higher contents of lignin and phenolics as compared with the grass leaves. During the first 2 years after litter fall decomposition of *Molinia* litter was on average four times faster than litter of *Erica* (Table 1). Decay rates of dead organic materials decrease rapidly with increasing age, meaning that the young litter cohorts will have an important impact on total soil nutrient mineralization as compared with the great bulk of old organic matter in the soil with a very low turn-over. Differences in fresh litter decay were found to have significant effects on soil nutrient mineralization rates. After 4 years, mineralization rates in garden plots that had been planted with monocultures of *Molinia caerulea* were almost twice as high as in the plots planted with dwarf shrubs (Berendse 1998). Clearly, plant features relevant to the adaptation of plant species to fertile or less fertile substrates have at the same time strong effects on soil nutrient availability.

The important question is whether plant-soil interactions can indeed result in positive feedbacks that lead to a runaway process and a strongly accelerated change in species composition. Intuitively, it seems plausible that plant species that have a positive effect on soil nutrient supply and can profit rapidly from an increased nutrient supply will strongly accelerate their expansion as soon as they have reached a certain critical abundance. However, it is extremely difficult to obtain empirical proof for this phenomenon.

The rapid shifts in plant dominance in heathlands that we described took largely place between 1975 and 1985. During the preceding decades the grass species had always been present, but only locally and at a low abundance. Model simulations showed that after nutrient mineralization had increased to sufficiently high levels the grasses expanded very rapidly, strongly promoted soil nutrient release and replaced the dwarf shrubs within < 10 years (Berendse 1998). The large scale replacement of heather by grass-dominated vegetation was triggered by an increase in atmospheric N-inputs, but was strongly accelerated by the effects that the grasses had on soil nutrient release. In the field these effects cannot be separated, but model simulations clearly show that without interspecific differences in litter decomposability, these changes in species composition take place much more slowly (Berendse 1994b). The presented arguments in isolation do not provide sufficient proof, but it is the combination of field observations of long-term vegetation changes, measured plant features, measured effects of these species on soil nutrient mineralization in garden plots and model simulations that provides convincing evidence that such runaway processes take place.

## Plant traits and nutrient availability

The differences between grasses and ericaceous dwarf shrubs mirror a much broader pattern among vascular plant species. Díaz *et al.* (2004) analysed 12 plant traits in 640 plant taxa occurring in four countries (England, Spain, Argentina and Iran) with extremely different climates and environments. In each of these countries, the same axis with at the one end thin leaves with high specific leaf area (SLA) and species with tough leaves with low SLA at the other end explained most of the variance. This axis represents precisely the difference between the ericaceous dwarf shrubs and the grasses in Dutch heathland, but also the difference between angiosperms and gymnosperms. It reflects the fundamental trade-off between a set of plant attributes that allow rapid acquisition of resources and fast growth and another set that enables conservation of resources within well-protected tissues (Grime 2002). Apparently, there are severe constraints that limit the possibilities to combine the features of both ends of this axis.

The plant features that dominate this axis are also strongly correlated with the decomposability of the litter that these species produce (Cornwell *et al.* 2008). This relationship is not surprising since high lignin contents enable long life spans of plant organs, but also slow down the microbial decay of these organs after their death. A recent meta-analysis showed that such plant features have stronger effects on litter decomposition rates than environmental variables such as precipitation and temperature. On average, studies comparing the litter decomposability of large numbers of species found a 18.4-fold difference in mass loss rate while holding climate constant (Cornwell *et al.* 2008), whereas experiments that incubated standard substrates in extremely different environments varying from tropical rainforest to boreal forest and tundra found a 5.5-fold (Parton *et al.* 2007) and a 5.9-fold (Berg *et al.* 1993) range in the rate of mass loss across sites.

Thus, low litter decomposability is in the first place a consequence of plant features that are favoured by direct natural selection in stressful, nutrient-poor environments. But variation in litter decomposability can also develop due to indirect, divergent selection through the different effects that plants have on soils. Using a model with two competing plant species that only differed in relative biomass loss and potential growth rate, we included the interaction with the soil substrate. Both species affected this substrate by producing litter, while their growth was affected by the amounts of nutrient released from the decomposing organic materials. In the slow growing species with a low biomass turnover fitness was increased by reducing litter decomposability, while in the fast growing species with a high biomass loss fitness increased when litter decomposability was increased (Berendse 1994b). These simulations suggest that in species showing correlated variation in growth rate and leaf life span, litter decomposability correlates as well not only because of the direct effects of plant features (such as lignin content) that influence litter decomposition, but also due to indirect selection acting by the interaction between plants and soils. The general and strong correlation between leaf life span (i.e. biomass turn-over) and litter decomposability has enormous – and often underestimated – consequences for the impact of plant species on soil nutrient release. Indeed, the combined effects of biomass turn-over and decomposability exceed by far the effects of these plant features in isolation (Berendse 1998). The general trade-off between high growth rates and protection of absorbed nutrients leads to much stronger impacts of dominant plant species on soil nutrient mineralization than we would expect if these plant features were randomly distributed over species.

## Another example: the expansion of dwarfshrubs and graminoids in bog ecosystems

Our hypothesis is also well in line with recent observations on critical transitions described for raised bogs. These peatmoss systems can shift to an angiosperm dominated state as atmospheric nitrogen input increases beyond a critical level. Peat mosses are able to grow in extremely nutrient-poor, wet, acidic conditions, where they are competitively superior with respect to most vascular plant species (van Breemen 1995). They are able to maintain these conditions by producing dead material that decomposes at very slow rates due to sphagnan (a pectin-like polysaccharide) and sphagnic acid (*p*-hydroxy-β-carboxymethyl-cinnamic acid) in their tissues and the anoxic, water-saturated conditions (Bartsch & Moore 1985;Verhoeven & Toth 1995). The dead materials that *Sphagnum* species produce decompose at much lower rates than the litter of the vascular plant species in these systems (Verhoeven & Liefveld 1997). Under these circumstances roots of vascular plants are only able to absorb very small amounts of mineralized nutrients, while the peatmosses primarily depend on the atmospheric deposition of nutrients. These conditions are stable as long as the system is water-saturated and nutrient inputs do not increase. But rapid transitions to another state can take place when nutrient inputs increase or geohydrological conditions change. Once nitrogen inputs exceed a critical level, the peat moss biomass becomes nitrogen saturated, after which part of the inorganic nitrogen input accumulates as ammonium in the interstitial water (Limpens *et al.* 2003; Tomassen *et al.* 2004). This accumulating inorganic nitrogen can be easily utilized by shallow rooting vascular plants that are already present in the system, such as *Vaccinium oxycoccus* or *Rhynchospora alba* (Limpens *et al.* 2004). As a consequence these species rapidly expand as has often been observed, both in nutrient addition experiments and in N-polluted bogs. When vascular plants have reached a certain critical abundance, their more easily decomposable litter increases N supply, which will promote further expansion of graminoids and dwarf shrubs. Moreover, the shadow of the vascular plant leaves and litter will limit peatmoss growth. Growth and nitrogen absorption in peatmosses that experience lower light intensities will be reduced so that the accumulation of ammonium in the interstitial water accelerates, leading to a positive feedback and a rapid expansion of vascular plants and finally to the establishment of birch trees and the development of woodlands.

## Final remarks

Summarizing, we propose that just like modern gymnosperms, the ancient gymnosperms – and especially the dominant conifers – could thrive at low nutrient supplies, but also kept nutrient availability in the soils low, as a result of the kind of litter they produced. A gymnosperm-dominated vegetation would thus represent a stable state, in which angiosperms could only enter once a critical soil nutrient supply was exceeded. This may explain, why the early angiosperms could only enter at sites without existing vegetation. However, as suggested by our modern case studies and models, once angiosperms had reached a certain critical abundance, they might have promoted higher nutrient levels in the soil to the point that a shift to the alternative stable state occurred. In the Cretaceous, long-distance seed dispersal of angiosperms from their strongholds may have enabled them to gradually take over adjacent habitats, previously dominated by gymnosperms. Spatial models of competition with alternative stable states show that such domino effects can occur (van Nes & Scheffer 2005). One can thus imagine a gymnosperm dominated world in which angiosperms had been suppressed for a long time. However, once unleashed they would become rapidly dominant in a positive-feedback cycle.

Clearly angiosperms would first have to reach a certain minimal abundance, before the proposed plant–soil feedbacks could have substantial consequences. So, an important yet unresolved question remains what may have triggered the initial expansion of the angiosperms? A large complex of factors may have played a role in facilitating early angiosperm development (e.g. Regal 1977; Bakker 1978; Barrett & Willis 2001). The mid- and Late Cretaceous radiations of flower visiting insects and seed dispersing animals may have facilitated angiosperm diversification and expansion, especially in areas where angiosperms still occurred in spatially isolated populations (Regal 1977; Mulcahy 1979; Barrett & Willis 2001). Nitrogen fixation may also have played a role. Symbiotic associations with cyanobacteria are found in some gymnosperm groups (cycads), but associations with rhizobia and Frankia occur only in nine extant angiosperm families and not in gymnosperms and ferns (Sprent 2005). The first angiosperms that could profit from gaseous N-fixation were probably able to expand rapidly in a nitrogen-poor world and perhaps even increased soil nitrogen levels over larger areas, although it should be noted that many angiosperm taxa did not have nitrogen-fixing capacities. Almost all angiosperm N-fixers belong to the fabids which appeared by the late Cenomian (95 Ma). So, angiosperm N-fixation could not have been a substantial factor until some time in the Late Cretaceous. Another hypothesis is that the shift from high-browsing sauropod dinosaurs in the Late Jurassic to the low-browsing ornithischian dinosaurs strongly increased the mortality of gymnosperm saplings and created disturbed environments from which gymnosperms were excluded due their low tolerance to herbivory (Bakker 1978). Although this sounds plausible, there is no convincing evidence in the fossil record for correlations between major events in the evolution of herbivorous dinosaurs and the origin of angiosperms (Barrett & Willis 2001). Another idea is that peaking CO_2_ levels in this period may have played a role (Barrett & Willis 2001). Elevated CO_2_ would have allowed higher water use efficiency and enabled plants to colonize drier areas. The higher CO_2_ levels during the mid Cretaceous were probably due to a pulse of global tectonic activity suggesting that the initiation of early angiosperm expansion could have been boosted by a major geological event. On the other hand, during the Late Cretaceous and the Paleocene atmospheric CO_2_ concentrations declined again, while during this period angiosperms became dominant around the globe. This suggests that – after the initial expansion – other mechanisms further propelled the developments and stabilized global angiosperm dominance. We would argue that the feedback between vegetation and soil fertility is an obvious and strong candidate. While minimal models may illustrate the point, the case for this idea is especially convincing in view of the insights obtained from controlled ecological experiments in combination with well studied recent shifts in vegetation dominance driven by the same mechanism we propose as the driver behind an ancient runaway shift from gymnosperms to angiosperms.

Obviously, inferring mechanisms behind events that happened in the deep past remains challenging. Nonetheless, our hypothesis implies some specific predictions that could be tested further by scrutinizing the geological record. First, the shift to angiosperms should coincide with increasing soil fertility which may be checked if appropriate proxies can be found. Second, the validity of the idea that angiosperm and gymnosperm dominance could represent alternative stable states may be explored in more detail by checking for the classical hallmarks of bistability (Scheffer & Carpenter 2003). In addition to the observed shift in dominance and the existence of a plausible positive feedback, these hallmarks include among other things the existence of a bimodality in the frequency distribution of states (dominance by either group being more common than balanced mixes). The probability of finding a particular group dominating should then depend on overall conditions. For instance, since modern as well as ancient gymnosperms appear to thrive relatively better in colder climates, we should expect to see mostly gymnosperm dominance at higher latitudes vs. angiosperm dominance at lower latitudes. At intermediate conditions one should expect a spatial patchwork with either of the groups dominating. While hard proofs of what caused a particular pattern in the far past will remain illusive, it should be feasible to mine the geological record for such more specific hallmarks associated with our suggested ecological explanation for Darwin’s abominable mystery.
